# Interplay Between Ion Channels and the Wnt/β-Catenin Signaling Pathway in Cancers

**DOI:** 10.3389/fphar.2020.525020

**Published:** 2020-09-29

**Authors:** Raphael Rapetti-Mauss, Camille Berenguier, Benoit Allegrini, Olivier Soriani

**Affiliations:** Inserm, CNRS, iBV, Université Côte d’Azur, Nice, France

**Keywords:** signaling pathways, drug targets, KCNQ1 channel, P2X7, cystic fibrosis transmembrane conductance regulator, hERG, transient receptor protein channels, ClC-2 channel

## Abstract

Increasing evidence point out the important roles of ion channels in the physiopathology of cancers, so that these proteins are now considered as potential new therapeutic targets and biomarkers in this disease. Indeed, ion channels have been largely described to participate in many hallmarks of cancers such as migration, invasion, proliferation, angiogenesis, and resistance to apoptosis. At the molecular level, the development of cancers is characterised by alterations in transduction pathways that control cell behaviors. However, the interactions between ion channels and cancer-related signaling pathways are poorly understood so far. Nevertheless, a limited number of reports have recently addressed this important issue, especially regarding the interaction between ion channels and one of the main driving forces for cancer development: the Wnt/β-catenin signaling pathway. In this review, we propose to explore and discuss the current knowledge regarding the interplay between ion channels and the Wnt/β-catenin signaling pathway in cancers.

## Introduction 

Despite decades of intensive research on its physiopathology, cancer remains a leading cause of death worldwide according to the World Health Organisation. Traditional cancer treatments including radiation therapy, chemotherapy, and surgery have substantially improved patients’ conditions. However, several challenges in curing cancer remain in front of us. Side effects generated by cancer treatments significantly impact patients quality of life; high patients intrinsic heterogeneity revealed treatments resistance, and finally the efficiency of treatment used for some types of cancer remains very low such as for pancreatic cancer. These circumstances highlight the urgent need to identify new therapeutic strategies in treating cancers.

Ion channels are transmembrane proteins allowing ions to cross biological membranes thereby generating electrical current and variation in membrane potential. Ion channels are classified in five main families, Potassium channels, Chloride channels, Sodium channels, Calcium channels that are selective for their specific ion, and non-selective ion channels that allow the passage of several species of ions. The functions attributed to ion channels were first confined to cell excitability and regulation of hydro-electrolytic balance. Interestingly, since the 1980’s ion channels have also been described to be involved in controlling processes regulating cell behaviors such as proliferation, migration, invasion and differentiation. In 1984, the pioneer work of DeCoursey describing the involvement of potassium channels in the proliferation of T lymphocytes (DeCoursey et al., 1984) opened new perspectives in studying the biology of ion channels. The first evidence for such functions of ion channels in cancer cells emerged in the late 1980’s with studies describing that the pharmacological inhibition of ion channels reduces tumor cells proliferation (Yamashita et al., 1987; Lee et al., 1988; Pancrazio et al., 1989; Taylor and Simpson, 1992). Since then, the field has greatly expanded, and it has been shown that ion channels are involved in virtually all the hallmarks of cancers described by Hanahan et al. (Prevarskaya et al., 2010; Prevarskaya et al., 2018). Although the involvement of ion channels in cancer has been clearly described, how ion channels interact with cancer-related signaling pathways from a mechanistic point of view, and how the expression of ion channels is regulated in cancers is poorly understood. However, this issue has recently emerged in the literature and a growing number of reports tackle these questions. Cancer initiation and development is often associated to altered signaling pathway involved in cell and tissue homeostasis (Sanchez-Vega et al., 2018). Among these signaling pathways, the Wnt/β-catenin pathway emerged as the most associated pathway to ion channels in the literature. This review aims to summarize the current knowledge regarding the interplay between ion channels and the Wnt/β-catenin signaling pathway.

## The Wnt Signaling Pathway

Since the first description of Wingless Int 1 (Wnt1) gene in virally induced breast tumors (Nusse and Varmus, 1982), the Wnt signaling pathway has been subjected to intense research, ranging from development to cancer. Its role in morphogenesis and adult stem cell renewal is closely linked to its involvement in cancer development (Nusse and Clevers, 2017). Mechanistically, Wnt signaling pathway can take mostly two directions: the canonical (Wnt/β-catenin), influencing cell-fate, proliferation, as well as self-renewal and the non-canonical (β-catenin-independent), Wnt/PCP, Wnt/Ca^++^, Wnt/STOP, influencing planar polarity (Tree et al., 2002), Ca^2+^ homeostasis (Sheldahl et al., 2003), or protein stabilization (Davidson et al., 2009), respectively. Both are involved in cancer but canonical Wnt/β-catenin remains the most studied in a large variety of tissues.

In mammals, there are more than 19 Wnt ligands which are expressed in a tissue-specific manner. After their translation into proteins Wnt ligands are modified with attachment of a palmitoleic acid (palmitoylation) by the ER-resident acyl-transferase Porcupine (Kadowaki et al., 1996). This modification allows binding of Wnt ligand to the ER-transmembrane protein Evi/Wls, and therefore the transport to Golgi apparatus (Bänziger et al., 2006) assisted by the p24 protein (Buechling et al., 2011). Wnt ligands are either released by solubilisation (Mulligan et al., 2012), by exosomes (Gross et al., 2012), or in lipoproteins particles (Neumann et al., 2009) or linked to the plasma membrane. Importantly, after being secreted, Wnt ligands can form a gradient and act as directional growth factor, as it is the case in intestinal colonic crypts (Medema and Vermeulen, 2011)

Here we will focus on Wnt canonical pathway, which depends on β-catenin stability. In the absence of Wnt ligand (Wnt OFF state), cytosolic β-catenin is sequestrated by a destruction complex (DC) composed of the tumor suppressor Axin acting as scaffold protein, the tumor suppressor Adenomatous Polyposis Coli (APC), the glycogen synthase kinase 3β (GSK3β) and casein kinase 1α (CK1α). Inside this DC, β-catenin is bound to Axin and phosphorylated by the two constitutively active kinases GSK3β and CK1α at specific Serine and Threonine residues (Liu et al., 2002). Then, β-catenin is ubiquitylated by the F-box containing protein E3 ubiquitin ligase (β-TrCP) and addressed to the proteasome for degradation (Aberle et al., 1997; Latres et al., 1999).

Initiation of the Wnt canonical (β-catenin-dependent) pathway (Wnt ON state) is mediated by engagement of Frizzled (Fz) receptor and LRP5/6 co-receptor with Wnt ligands. Activation of the pathway leads to degradation complex relocation near Fz/LRP complexes, where LRP is phosphorylated by CK1α and GSK3β. The phosphorylation of LRP triggers Dishevelled (Dvl) protein polymerisation, thus inactivating the degradation complex (Metcalfe et al., 2010). Another study suggests a direct inhibition of GSK3β by the phosphorylated-LRP co-receptor (Stamos et al., 2014). Hence, β-catenin is not degraded, can accumulate in the cytoplasm and translocate into the nucleus. β-catenin then complexes with lymphoid enhanced factor (LEF) and T-cell factor (TCF) to act as a transcription factor activating target genes (Li et al., 2012). In the Wnt OFF state, TCF interacts with Groucho, preventing transcriptional activation of Wnt target genes (Cavallo et al., 1998). Interestingly, β-catenin plays a second role in maintaining epithelial junctions, by binding to the cytoplasmic tail of E-cadherin (Peifer et al., 1992). Thus, presence of epithelial junctions is a limiting factor for Wnt/β-catenin activity, and reciprocally (Huels et al., 2015).

The Wnt/β-catenin pathway is tightly regulated. For example, the two homologues E3 ubiquitin ligases ZNRF3/RNF43 can induce Fz receptor lysosomal degradation, thus inhibiting Wnt signaling. R-spondins are secreted proteins, interacting with their receptor LGR4-6, able to inhibit ZNRF3/RNF43 activity (de Lau et al., 2011). Furthermore, Hippo and Wnt pathway have been closely linked, since the Hippo transducer YAP/TAZ plays an integrative role inside the DC, allowing β-TrCP recruitment to the complex and subsequent β-catenin degradation (Azzolin et al., 2014). Others regulations are nicely reviewed by Zhan et al. (2017).

As mentioned above, Wnt canonical pathway is widely implicated in cancer diseases. The first mutations have been found in the APC gene, leading to Familiar Adenomatous Polyposis (Kinzler et al., 1991; Nishisho et al., 1991), and now found in the majority of colorectal cancers (Wood et al., 2007). Mutations of Axin1 are present in hepatocellular carcinoma (Satoh et al., 2000). Gain of function β-catenin mutations are reported in melanoma (Rubinfeld et al., 1997). Loss of function mutations in Rnf43 or Znrf3 are found in pancreatic cancer (Wu et al., 2011) and adrenocortical carcinoma (Assié et al., 2014). Wnt signaling has also been implicated in cholangiosarcoma (Chan-On et al., 2013), leukemia (Wang et al., 2010) and in 50% of breast cancer cases (Lin et al., 2000).

## Wnt Signaling Pathway Interplay With Potassium Channels

With more than 80 members, the potassium channel family is the widest group of ion channels. They participate in a multitude of biological processes from cell excitability to water and electrolyte homeostasis. They are especially essential in maintaining membrane resting potential. Increasing evidence point out the involvement of this family of ion channel in the physiopathology of cancers (Huang and Jan, 2014; Pardo and Stühmer, 2014). However, the molecular mechanisms sustaining potassium channels involvement in cancer are still unclear. This section reviews recent work that report the function of potassium channels in the Wnt/β-catenin pathway.

## KCNQ1

The potassium channel KCNQ1, also called Kv7.1, belongs to the family of voltage-gated potassium channel. It is constituted by the assembly of four α subunits forming the pore of the channel. This channel can associate with ancillary subunits from the KCNE family which drastically change the channel features regarding the KCNE associated (Bendahhou et al., 2005; Jespersen et al., 2005). Association with KCNE1, shows a delay in KCNQ1 activation and in this case the complex KCNQ1/KCNE1 also called IKs, is essential to cardiac action potential repolarisation. With KCNE3 and KCNE2, KCNQ1 is no longer voltage-dependent and participates to transepithelial transport in nearly every epithelium. The effect of the last two members of the KCNE family on KCNQ1 are more discussed in the literature, however it appears that KCNE4 and KCNE5 mostly act as inhibitors of KCNQ1 activity (Abbott, 2014).

As aforementioned, KCNQ1 plays a key role in the physiology of epithelial transport. Transport mechanisms involve complex networks of ion channels and transporters, creating and dissipating ionic gradients required for the passage of water, electrolytes, nutrients or other substrates through the epithelium (Barrett and Keely, 2000). Thus, the Na^+^/K^+^ ATPase pump activity is the main force for transepithelial transport allowing Na^+^ output against K^+^ entry into the cell. In this context, K^+^ channels provide two main functions: the maintenance of membrane potential and the basolateral recycling of K^+^ that guarantees the maintenance of electrochemical gradients. In the colon, KCNQ1, associated with its regulatory subunit KCNE3, plays a fundamental role in Leiberkühn’s crypts homeostasis by maintaining the electrical driving force required for apical secretion of Cl^-^ and water, and Na^+^ absorption (Preston et al., 2010; Rapetti-Mauss et al., 2013).

Recently, studies found an unexpected link between KCNQ1 and epithelial cancers. In a first study, using mouse models, the authors have shown that the loss of KCNQ1 expression promotes the development of adenocarcinoma in the colon. They also showed that the low expression of KCNQ1 is associated with a poor survival prognosis in patients with Colorectal Cancer (CRC) showing hepatic metastases (Than et al., 2014). In line with these findings, another report has shown that in patients with stage II and III colon cancer, low expression of KCNQ1 is associated with poor survival prognosis. This channel therefore seems to be an interesting prognostic marker for the early stages of the disease as well as for the prediction of recurrences (den Uil et al., 2016). These studies also suggest that KCNQ1 acts as a tumor suppressor in CRC. Another study described the same function of KCNQ1 in Hepatocellular Carcinoma (HCC). They have shown using immunohistochemistry that HCC patients’ samples show less KCNQ1 expression compared with normal liver tissues. Also, in patients with HCC, the high expression of KCNQ1 significantly correlates with a better patient’s survival rate. Therefore, they reach the same conclusion as in CRC, KCNQ1 acts as a tumor suppressor in HCC (Fan et al., 2018).

It is clearly not trivial to explain the function of KCNQ1 as a tumor suppressor protein in epithelial cancers by its classical role in the physiology of epithelial transport. Therefore, could alternative functions of this channel underlie the observed correlations between KCNQ1 expression and cancers development? We and other have explored this issue to identify the molecular mechanism sustaining the tumor suppressor functions of KCNQ1 in CRC and HCC.

It appears that KCNQ1 expression is associated in CRC and HCC to epithelial cell plasticity (Rapetti-Mauss et al., 2017; Fan et al., 2018). Epithelial-to-Mesenchymal Transition (EMT) is a cellular process by which epithelial cells progressively and reversibly acquire mesenchymal features (Ye and Weinberg, 2015; Pastushenko and Blanpain, 2019). This process has largely been associated to cancer development and progression by allowing loss of epithelial structure and promoting cancer cell migration, invasion and drug resistance. In CRC, the expression of KCNQ1 in a panel of cell lines showing different degrees of EMT is associated with epithelial features. Conversely, the channel’s expression is lowered in most mesenchymal cell lines. These data raise the hypothesis of a functional link between KCNQ1 and the cellular process of epithelial-to-mesenchymal transition in cancer cells. Interestingly, in CRC as well as in HCC, the rescue expression of KCNQ1 in cell lines that do not express this channel, restores the expression of epithelial markers such as E-cadherin, lowers the expression of mesenchymal markers such as N-cadherin and restores the ability of cell to form spheroids in 3D culture. Moreover, the molecular silencing of KCNQ1 in epithelial CRC cell line reduces the expression of E-cadherin, increases the expression of N-cadherin and impairs the ability to grow as a spheroid in 3D culture. Therefore, the expression of KCNQ1 seems to promote epithelial features.

An important event characterising EMT is the dislocation of adherens junctions (AJ) causing the loss of cohesion between adjacent cells and therefore disrupting the epithelial integrity. The exploration of intestinal crypts by techniques coupling immunocytochemistry and in-situ immunisation (proximity ligation assay), provides data showing that KCNQ1 is directly associated with the β-catenin/E-cadherin complex in AJ in colonocytes. Interestingly, the same association between KCNQ1 and β-catenin has been established in hepatocyte cell lines. At the functional level, silencing of KCNQ1 expression in colon cell lines produces a disruption of the complex, as well as a loss of the integrity of epithelial architecture, which results in a decrease in epithelial electrical resistance. The study of the phosphorylation state of β-catenin in cell lines where KCNQ1 has been silenced by shRNA, indicates that KCNQ1 is necessary to retain β-catenin at the plasma membrane. Beside this observation it has been shown that pharmacological or molecular inhibition of KCNQ1 in CRC and in HCC, leads to a potentiation of the Wnt/β-catenin signaling pathway. Indeed, silencing of KCNQ1 in CRC induces the re-localisation of β-catenin in the cytosol and increases the expression of Wnt/β-catenin signaling pathway targets such as Cyclin D1 and C-jun. In HCC overexpression of KCNQ1 reduces Wnt target genes expression and Wnt activity using a Top/fop assay. These results indicate that KCNQ1 restricts the Wnt/β-catenin signaling pathway activity, stabilising β-catenin at the plasma membrane. These results reveal a new role for KCNQ1: in parallel with its function of transepithelial transport regulator, this channel plays a predominant role in maintaining the architectural integrity of the epithelium. These results shed new light on the correlation between the level of KCNQ1 expression and the clinical outcome of patients with CRC, since high levels of channel expression, together with the maintenance of adherens junctions guarantee the sequestration of β-catenin to the membrane, repression of the Wnt/β-catenin pathway, and finally reduces the commitment in the EMT process.

The stabilisation of the β-catenin/E-cadherin/KCNQ1 complex at the AJ constitutes an explanation to understand the role of KCNQ1 as a tumor suppressor in CRC and HCC. Would the latter only play an anchoring protein role for the β-catenin/E-cadherin complex, or is the KCNQ1-induced hyperpolarising potassium flow important in the stabilisation of the complex? In other words, do membrane potential variations influence β-catenin localisation and activity? The answer is provided by experiments in which CRC cells expressing KCNQ1 are experimentally depolarised by increasing the extracellular K^+^ concentration 140mM K^+^. This protocol causes a cytosolic accumulation of β-catenin, suggesting that the depolarisation induced by the silencing of the channel potentiates Wnt/β-catenin pathway. These results demonstrate the existence of a link between the electrical signature of the cancer cell and the signaling pathways that control cancer cells features. These data are consistent with a recent study that shows that the K-Ras/RAF/MAPK signaling pathway is directly controlled by membrane potential (Zhou et al., 2015).

There is only few information in the literature regarding how ion channel expression is regulated in the context of cancers. It has been observed that expression of KCNQ1 is decreased in both CRC and HCC. We have tempted to address this question and we reason that the link between KCNQ1 could be bidirectional. Is KCNQ1 expression repressed by Wnt activity? When the Wnt/β-catenin pathway is experimentally stimulated by pharmacological or molecular ways, we observed a decrease in KCNQ1 expression. We also identify that β-catenin as well as the TCF-4 transcription factor (member of the TCF/LEF family, which regulates the expression of the target genes of the Wnt pathway), bind directly to KCNQ1 promoter and cause the repression of its expression. These results thus reveal that KCNQ1 channel is a new target gene of Wnt pathway (Rapetti-Mauss et al., 2017).

By unveiling the bidirectional interaction between Wnt/β-catenin pathway and KCNQ1, these works reveal new functions of potassium channels in healthy tissue architecture, and their implication in the development of tumors. Thus, the fine understanding of these alternative functions could contribute to the characterisation of new markers, and innovative therapeutic targets. These data suggest that KCNQ1, by sequestering β-catenin at the AJ, restricts activation of Wnt signaling pathway and acts as a tumor suppressor in multiple epithelial cancers. The mechanism seems to be quite conserved suggesting a key role of KCNQ1 in epithelial homeostasis.

## KCNH2

Another potassium channel has recently been associated to the Wnt/β-catenin pathway, KCNH2 also called hERG or Kv11.1 (Breuer et al., 2019). This channel is a voltage-gated potassium channel, which role in cancer cells has largely been studied especially by the groups of Arcangeli (Becchetti et al., 2019) and Soriani (Crottès et al., 2016). This channel is associated with β1-integrin when the channel is in its closed state, resulting in an increase of metastasis in breast cancer. Whereas, in the open state, the channel association with β1-integrin is decreased leading to a reduction in breast cancer metastasis (Becchetti et al., 2017).

A recent report described that an opener of hERG, the NS1643, reduces metastasis spread of breast tumor *in vivo* by attenuation of the Wnt/β catenin pathway signaling, suggesting that NS1643 mimics the dissociation between hERG and β1-integrin (Breuer et al., 2019). The authors observed that activating hERG channel stabilises β-catenin at the plasma membrane by inhibiting AKT (T308) and GSK3β (S9) phosphorylation. In this case the cytosolic fraction of β-catenin decreases favouring the association of β-catenin with E-cadherin. Therefore, NS1634 decreases Wnt pathway activity. This result suggests that the hyperproliferation induced by the opening of hERG reduces Wnt activity by retaining β-catenin at the plasma membrane. These data are consistent with the data from Arcangeli’s lab showing that the open state of hERG lowers metastasis dissemination in breast cancer.

## Wnt Signaling Pathway Interplay With Chloride Channels

Chloride channels regulate a variety of cell processes such as excitability and water and mucus secretion. Another important aspect of these channels, especially in cancer, is their role in cell volume regulation and pH level regulation. As for the other ion channel families, different chloride channels have been described depending on their gating properties: calcium-activated, voltage-dependant, ligand-dependant. Although their role in cancer has been under study for the past years, their link with the Wnt/β-catenin pathway remains poorly investigated.

## CFTR

Cystic Fibrosis Transmembrane Conductance Regulator (CFTR) is widely expressed in the apical epithelial membranes. CFTR is an anion channel regulating fluid transport and pH levels. Mutations of CFTR such as ΔF508 result in Cystic Fibrosis (CF) causing a wide range of symptoms linked to a defect in fluid transport across the epithelial membranes. The pulmonary symptoms are well-described, but the earliest symptoms concern the digestive system. In addition, CF patients are at high risk of developing gastrointestinal cancers (Yamada et al., 2018). The study from Strubberg et al. links for the first time the loss of functional CFTR and gastrointestinal cancer through the increase of the proliferation rate of crypt cells by activating Wnt/β-catenin pathway (Strubberg et al., 2018). Indeed, the authors show that loss of functional CFTR leads to an increase of intracellular pH (pHi) levels in crypt cells as Cl^-^ and HCO3^-^ secretion is impaired. In addition, it has been shown in *Drosophila* species that alkaline pHi (negatively charged phospholipids at the inner leaflet) facilitates Dishevelled’s interaction with the Wnt receptor Frizzled by addressing Dishevelled to the negatively charged phospholipids through its DEP domain (Simons et al., 2009). Strubberg et al. show that the same mechanism is occurring in Cftr KO cells. The inhibition of CFTR activity alkalinizes pHi in the crypt epithelium, enhancing Dishevelled-2’s (Dvl-2) association at the plasma membrane and promoting binding between Fz and Dvl-2 and therefore facilitating Wnt signaling. Confirming these results, active β-catenin and LEF1 levels were higher in Cftr KO mice crypts than WT. Furthermore, CFTR has been shown to be a direct target of Wnt signaling as it exhibits an intestine-specific enhancer element positively regulated by TCF4 (Paul et al., 2007). All in all, loss of CFTR facilitates Wnt signaling in the intestinal crypt epithelium, increasing tumorigenesis and underlying the basis of increased risk of gastrointestinal cancers in CF patients.

The main cancer risk for CF patients is gastrointestinal (GI) cancers (Neglia et al., 1995). However, as new therapeutic strategies aim to enhance CF patients’ life expectancy, and since the link between loss of functional CFTR and Wnt/β-catenin pathway has been assessed in GI cancers, other epithelial cancers could occur in CF patients following the same biological process.

## ClCN2

Adherens junctions and tight junctions ensure epithelium integrity, providing a physical barrier. Adherens junctions further establish the apical-basal cell polarity. The importance of the chloride channel ClCN2, also called ClC-2, in the regulation of tight junctions’ composition in the small intestine has been recently uncovered (Nighot and Blikslager, 2012). As ClCN2 seemed to be an important actor of epithelial integrity and maintenance of the tight junctions, Jin *et al*. questioned its involvement in adherens junctions (AJ) and its interaction with β-catenin (Jin et al., 2018). β-catenin binds E-cadherin in the adherens junctions. If the AJ are disrupted, β-catenin is freed from the membrane-bound E-cadherin, accumulates in the cytosol and translocates to the nucleus where it interacts with TCF/LEF1, activating the Wnt target genes.

ClCN2 is expressed physiologically in the colonic epithelium. Absence of ClCN2 alters AJ integrity, inducing an increase of nuclear β-catenin. The increase of nuclear β-catenin is linked to an upregulation of Wnt target genes. Moreover, the loss of ClCN2 alters colonic crypt homeostasis, increases proliferation and reduces differentiation of colonic cells.

Therefore, the loss of ClCN2 in colonic epithelium leads to the disruption of adherens junction and subsequent activation of Wnt pathway, promoting tumorigenicity. The involvement of other ClCs in the regulation of junctions and their link with Wnt/β-catenin pathway has not yet been assessed. However, a study showed that absence of a regulatory protein of Calcium-activated chloride channels (CaCCs)—such as ANO1, also called TMEM16A (Sala-Rabanal et al., 2015; Sala-Rabanal et al., 2017)—CLCA1, induces an increase of β-catenin in the nucleus (Li et al., 2017). However, this study only focuses on the consequence of a knock-out of CLCA1 and not its link with CaCC channels nor its direct or indirect interaction with Wnt/β-catenin pathway. These points remain to be understood to truly consider CLCA1 as a tumor suppressor *via* inhibition of Wnt pathway.

## Wnt Signaling Pathway Interplay With Other Channels

### TRP Channels

Transient receptor protein (TRP) channels are cationic channels that generate Na^+^ and Ca^2+^ influxes in response to a great variety of stimuli (Venkatachalam and Montell, 2007). Therefore, TRP channels enable individual cells to sense changes in their local environment, translating this information into signals that can be interpreted at a short time scale such as membrane potential and Ca^2+^ homeostasis (Shapovalov et al., 2016). Several works have revealed a functional link between TRP channels and Wnt/β-catenin pathway in cancers.

#### TRPV4

In gastric cancers, the Calcium receptor (CasR) is overexpressed. Its overexpression contributes to tumor growth, metastasis formation, and poor prognosis. Mechanistically, CasR stimulates TRPV4 channels. The TRPV4-dependent Ca^2+^ influx increases AKT activation, which in turn phosphorylates β-catenin on its residue Ser675 and thus activating β-catenin downstream signaling (Xie et al., 2017).

#### TRPM4

TRPM4 channels are overexpressed in prostate and colorectal cancers where they participate to migration and invasion (Gao and Liao, 2019). In prostate cancer cells, invalidation of TRPM4 increases GSK3β activity, leading to β-catenin degradation. In LnCAP prostate cancer cells, TRPM4-associated Ca^2+^ influx stimulates AKT1, which in turn increases the inhibitory Ser9 phosphorylation of GSK3β and the total amount of β-catenin (Sagredo et al., 2018). In this study, Sagredo et al. suggest that the effect of TRPM4 on AKT1 is probably mediated by an alteration in the calcium/calmodulin-EGFR axis, linking TRPM4 activity with the observed effects in β-catenin-related signaling pathways.

#### TRPM8

A recent study driven in prostate and breast cancer cell lines has proposed that Wnt ligand-induced signaling involves TRPM8 channels. The authors propose a model in which Wnt ligands bind to both their receptors and TRPM8 channels, inducing a rapid decrease in [Ca^2+^]i, followed by a store-operated calcium entry (SOCE). The SOCE in turn triggers calcium-dependent potassium channels (KCNN4) leading to a large membrane hyperpolarization. This study indeed suggests that control of membrane potential by Wnt ligands is an early event of the Wnt signaling pathway (Ashmore et al., 2019).

#### TRPC5

TRPC5 is overexpressed, together with ATP-binding cassette, subfamily 1-member1 (ABCB1) in 5-Fluorouracil (5-FU), in CRC cells. ABCB1 is a transport protein involved in chemoresistance by mediating efflux of cytotoxic drugs. Enforcing TrpC5 expression increases in a Ca^2+^-dependent manner nuclear accumulation of β-catenin and subsequent upregulation of Wnt target genes including ABCB1. These results suggest that TRPC5 may reinforce chemoresistance by regulating transporters controlled by the Wnt/β-catenin pathway (Wang et al., 2015).

### Purinergic Ionotropic Receptors

#### P2RX7

The ionotropic ATP receptor P2RX7, (P2X7) is overexpressed in osteocarcinoma and participates to several cancer hallmarks including proliferation, migration, invasion and EMT. At the molecular level, P2X7 stimulation by receptor agonist (bzATP) increases GSK3β phosphorylation and further accumulation of β-catenin in the nucleus. Indeed, Wnt/β-catenin activation by P2X7 stimulation depends on PI3K/AKT stimulation. Interestingly, pharmacological inhibition of P2X7 decreased pro-tumoral behavior of cancer cells (Zhang et al., 2019).

### Acid Sensing Ion Channels

**ASIC1a:** Acid sensing ion channels (Asic) are proton-gated Na^+^/Ca^2+^ channels involved in a wide range of physiological features including pain perception and behavior (Hanukoglu and Hanukoglu, 2016). Asic1a is up-regulated in liver cancer tissues and cells lines and is associated to poor prognosis. KO of Asic1a inhibits liver cancer growth *in vivo* and *in vitro*, an effect that is underlied by cell cycle arrest and increased apoptosis. Jin et al. observed that low extracellular pH occurring in tumor microenvironment, increased Asic1a activity, which in turn decreases the Ser33 phosphorylation of β-catenin, leading to its accumulation in the nucleus, its combination with LEF/TCF promoting target gene transcription associated to liver cancer proliferation (Jin et al., 2017).

## Conclusion and Future Directions

**Figure 1:**
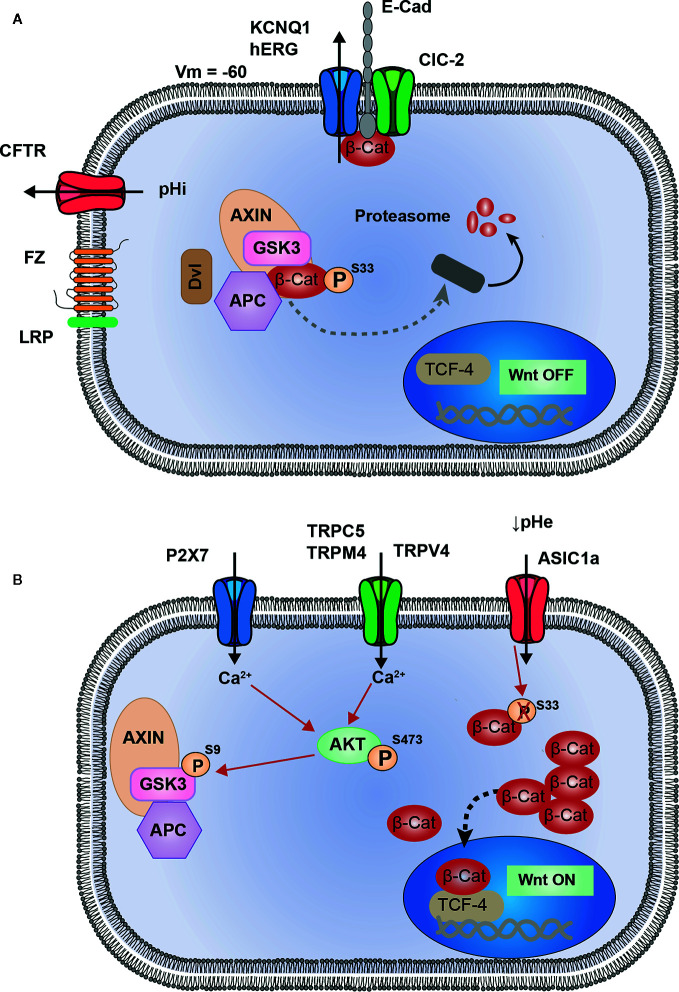
Proposed model of interactions between ion channels and Wnt signaling. **(A)** KCNQ1 (or hERG) and ClC-2 are associated to E-cadherin and β-catenin at adherent junctions (AJ). Activity of these two channels maintain membrane potential hyperpolarized, which could be a favorable condition for AJ stability, and sequestration of β-catenin at the plasma membrane. CFTR activity maintains intracellular pH, preventing from pHi alkalinisation which induces Dishevelled-dependant inhibition of Destruction Complex (DC). These four channels participate in maintaining Wnt signaling in OFF-state, in which β-catenin is either degraded or sequestrated and cannot translocate into the nucleus to induce Wnt target genes. **(B)** Ca^2+^ entry through TRPC5, TRPM4, TRPV4 and P2X7 channels, induces AKT activation. Thus, AKT phosphorylates GSK3-β which becomes inhibited and subsequently allows the cytosolic accumulation of β-catenin. ASIC1a, by maintaining an acidic pH, decreases β-catenin phosphorylation and ubiquitination, preventing its degradation and inducing a cytosolic accumulation.

In this review we have summarized the current work regarding the interplay between ion channels and the Wnt/β-catenin signaling pathway in cancers. The analysis of the literature highlighted 10 ion channels associated to the Wnt pathway (**Table 1**). Among these channels four are associated to the repression of Wnt pathway (KCNQ1, hERG, CFTR, and ClCN-2, **Figure 1A**). The loss of activity or expression of these channels leads to an increase of Wnt activity and cancer progression. Supporting this, KCNQ1, CFTR and ClCN-2 have been described as tumor suppressors in CRC. Contrarily, six ion channels are overexpressed in cancers and promote the activity of Wnt pathway (TRPC5, TRPV4, TRPM4, TRPM8, P2RX7, and ASIC1a, **Figure 1B**).

**Table 1:** Role of ion channels in regulating Wnt pathway activity in cancers.

(C) Cancers Type	Models	Channels	Effect of channels expression/activity on Wnt pathway	Mechanism	Expression in cancers	References
Colorectal Cancer	Cell lines, Rat colonic crypts	KCNQ1	↓ Wnt activity Wnt target genes	Stabilization of β-cat. at the plasma membrane	Down	Rapetti-Mauss et al., 2017
Hepatocellular Carcinoma	Cell lines	KCNQ1	↓ Wnt activity	Stabilization of β-cat at the plasma membrane	Down	Fan et al., 2018,
Breast cancer	Mouse models, Cell lines	hERG	↓ Wnt activity (Channel opened)	Stabilization of β-cat at the plasma membrane	Up	Breuer et al., 2019
Colorectal cancer	Mouse intestine	CFTR	↓ Wnt activity Wnt target genes	Modulation of the intracellular pH	Down	Strubberg et al., 2018
Colorectal cancer	Cell lines; Mouse models	ClC-2	↓ Wnt activity	Stabilization of β-cat at the plasma membrane	Down	Jin et al., 2018
Gastric cancer	Human sample, mouse model, Cell lines	TRPV4	↑ Wnt activity	Ca^2+^ → AKT → β-cat accumulation	Up	Xie et al., 2017
Prostate cancer	Cell lines	TRPM4	↑ Wnt activity	Ca^2+^ → AKT → β-cat accumulation	Up	Sagredo et al., 2018
Prostate cancer Breast cancer	Cell lines	TRPM8	↑ Wnt activity	Wnt ligands binding → calcium	Up	Ashmore et al., 2019
Colorectal cancer	Cell lines	TRPC5	↑ Wnt activity	Ca^2+^ → AKT → β-cat accumulation	Up	Wang et al., 2015
osteosarcoma	Cell lines	P2X7	↑ Wnt activity	AKT →GSK3 β → β-cat accumulation	Up	Zhang et al., 2019
Liver cancer	Human sample, Cell lines	ASIC1a	↑ Wnt activity	Modulation of β-cat phosphorylation and accumulation	Up	Jin et al., 2017

Several conclusions can be drawn from this analysis of the literature. Firstly, the hyperpolarisation induced by the opening of potassium channel (background current from KCNQ1/KCNE3 in colonocytes or inducing the opening of hERG in breast cancer cells) leads to the restriction of Wnt activity by a similar mechanism, i.e. the sequestration of β-catenin at the plasma membrane and probably at the adherens junctions. Hyperpolarised plasma membrane seems to be important to prevent an over activation of the Wnt pathway and stabilise β-catenin at the plasma membrane. Therefore, the remaining question is: how can membrane potential act on β-catenin stability at the membrane? This is an outstanding question which will need more investigation to be answered. Secondly, regarding the Wnt-activating ion channels, the mechanism emerging from this analysis seems to mainly involve an increase in intracellular [Ca^2+^] due to the overexpression or up regulation of cationic channels that mediate Ca^2+^ influx. The increase in intracellular [Ca^2+^] shunts the canonical Wnt/β-catenin signaling cascade by activating AKT. AKT, in turn phosphorylates GSK3β on S9 causing its inhibition and finally the accumulation of β-catenin and the activation of Wnt pathway. Thirdly, as mentioned above, several ion channels are involved in the regulation of Wnt pathway, suggesting that interaction between them could exist. This is true especially regarding the case of KCNQ1, CFTR, and ClCN-2. It is interesting to note that KCNQ1 and ClCN-2 share the same mechanism to lower Wnt pathway activity. Both channels are associated to adherens junctions and contribute to stabilise β-catenin at the plasma membrane. This observation suggests a functional interaction between these channels which has not been investigated so far. Concerning KCNQ1 and CFTR, it is largely described that in colonic epithelium both channels are closely linked in the mechanism regulating the transepithelial transport of Cl^-^ and water. It seems credible that this functional link is conserved in the regulation of the Wnt pathway, both channels contributing to restrict its activity by two different, but complementary mechanisms, KCNQ1 controlling the stability of β-catenin at the plasma membrane and CFTR controlling the localization of Dvl. It will be interesting to study the effect of the simultaneous inhibition of both channels on Wnt activity.

Large research efforts have been made during the last 40 years to describe and understand the functions of ion channels in controlling cellular processes. Although the involvement of ion channels in processes participating in cancer development has largely been demonstrated, the precise mechanisms sustaining these abilities remain mainly unknown. Even if several functions of ion channels in signaling pathways can be explained by classical roles, such as regulation of cell volume or modulating the intracellular calcium concentration, other implication of this class of proteins are dictated by original mechanisms. Could ion channels be necessary in the regulation of classical signaling pathway and how? Can we insert ion channels in the notebook picture of signaling pathways beside tyrosine kinases receptors and G protein coupled receptors? Answering these questions are the arising challenges in the field of ion channels in cancer biology.

## Author Contributions

RR-M conceived the presented idea and supervised the project. RR-M, CB, BA, and OS wrote the manuscript. All authors contributed to the article and approved the submitted version.

## Funding

This work was supported by the Foundation ARC (grant PJA 20181207701), “La Fondation pour la Recherche Médicale”—FRM—(PhD studentship to CB) (n° ECO201906008972) and the Cancéropole PACA (grant n° 2018-13) and Université Côte d'Azur (CSI-2018).

## Conflict of Interest

The authors declare that the research was conducted in the absence of any commercial or financial relationships that could be construed as a potential conflict of interest.
